# 

**Published:** 1889-12

**Authors:** 


					﻿DECEMBER, 1889.
OLD SERIES
Vol. XXV
NEW SFKitS/
Vol. VI -No. 10.'/
& 1855 O


(dHURNAk



W. F. WESTMORELAND, M.D.
H. V. M. MILLER, M.D., LL.D.
VIRGIL 0. HARDON, M.D.
F. W. McRAE, M.D,, Man. & Pro.
Published By james p.harrison&c®
£______________iflT LANTA, GA. JM
SUBSCRIPTION; S2.6O A
				

